# Applications of Artificial Intelligence-Based Systems in the Management of Esophageal Varices

**DOI:** 10.3390/jpm14091012

**Published:** 2024-09-23

**Authors:** Vlad Dumitru Brata, Victor Incze, Abdulrahman Ismaiel, Daria Claudia Turtoi, Simona Grad, Raluca Popovici, Traian Adrian Duse, Teodora Surdea-Blaga, Alexandru Marius Padureanu, Liliana David, Miruna Oana Dita, Corina Alexandrina Baldea, Stefan Lucian Popa

**Affiliations:** 1 Faculty of Medicine, “Iuliu Hatieganu” University of Medicine and Pharmacy, 400000 Cluj-Napoca, Romania; brata_vlad@yahoo.com (V.D.B.); turtoidariaclaudia@gmail.com (D.C.T.); adrianduse@yahoo.com (T.A.D.); alexandru.padureanu@outlook.com (A.M.P.); miruna.dita@outlook.com (M.O.D.); 2 2nd Medical Department, “Iuliu Hatieganu” University of Medicine and Pharmacy, 400000 Cluj-Napoca, Romania; abdulrahman.ismaiel@yahoo.com (A.I.); costinsimona_m@yahoo.com (S.G.); dora_blaga@yahoo.com (T.S.-B.); lilidavid2007@yahoo.com (L.D.); popa.stefan@umfcluj.ro (S.L.P.); 3 Faculty of Environmental Protection, University of Oradea, 26 Gen. Magheru St., 410087 Oradea, Romania; corina.baldea@uoradea.ro

**Keywords:** artificial intelligence, esophageal varices, variceal bleeding, endoscopy, CT scans, clinical data

## Abstract

Background: Esophageal varices, dilated submucosal veins in the lower esophagus, are commonly associated with portal hypertension, particularly due to liver cirrhosis. The high morbidity and mortality linked to variceal hemorrhage underscore the need for accurate diagnosis and effective management. The traditional method of assessing esophageal varices is esophagogastroduodenoscopy (EGD), which, despite its diagnostic and therapeutic capabilities, presents limitations such as interobserver variability and invasiveness. This review aims to explore the role of artificial intelligence (AI) in enhancing the management of esophageal varices, focusing on its applications in diagnosis, risk stratification, and treatment optimization. Methods: This systematic review focuses on the capabilities of AI algorithms to analyze clinical scores, laboratory data, endoscopic images, and imaging modalities like CT scans. Results: AI-based systems, particularly machine learning (ML) and deep learning (DL) algorithms, have demonstrated the ability to improve risk stratification and diagnosis of esophageal varices, analyzing vast amounts of data, identifying patterns, and providing individualized recommendations. However, despite these advancements, clinical scores based on laboratory data still show low specificity for esophageal varices, often requiring confirmatory endoscopic or imaging studies. Conclusions: AI integration in managing esophageal varices offers significant potential for advancing diagnosis, risk assessment, and treatment strategies. While promising, AI systems should complement rather than replace traditional methods, ensuring comprehensive patient evaluation. Further research is needed to refine these technologies and validate their efficacy in clinical practice.

## 1. Introduction

Esophageal varices are submucosal dilations in the lower esophagus, resulting from portal hypertension commonly seen in various liver and extrahepatic diseases, with liver cirrhosis being the most frequent cause [1]. The significance of proper diagnosis and treatment lies in the substantial morbidity and mortality associated with esophageal varices, particularly variceal hemorrhage. Key components of effective management include early and accurate identification of patients at high risk for variceal hemorrhage, selecting optimal treatment for each patient, managing acute variceal hemorrhage if it occurs, and secondary prophylaxis to prevent further bleeding [2,3].

Esophagogastroduodenoscopy (EGD) is currently the gold standard for assessing esophageal varices. It serves both diagnostic and therapeutic roles, enabling risk assessment for variceal hemorrhage and, when needed, performing elastic band ligation or sclerotherapy of the varices [4]. However, there are limitations to the primary methods for evaluating esophageal varices. EGD exhibits significant interobserver variability and relies on macroscopic characterization of the varices, based on their size and the presence or absence of warning signs indicating the risk of hemorrhage. Additionally, EGD is an invasive procedure, uncomfortable for the patient, and carries certain risks [5].

CT and MRI evaluations offer the advantage of visualizing the liver, portal venous system, and spleen anatomy. These non-invasive methods assess portal hypertension and esophageal varices, especially for identifying high hemorrhage risk [6,7]. However, these methods are expensive, with CT exposing patients to radiation and MRI being less available in many centers.

Recent studies have aimed to develop systems to quantify and classify the risk in patients with esophageal varices using clinical scores, laboratory data, or imaging, reducing the need for EGD [8,9]. Elastography is a crucial non-invasive assessment for liver cirrhosis patients, measuring liver stiffness and indirectly indicating portal hypertension, thus associated with the risk of esophageal varices [10].

The latest Baveno consensus guidelines recommend various laboratory parameters and clinical scores for evaluating and stratifying the risk of esophageal varices. These include the Child–Turcotte–Pugh (CTP) and Model for End-Stage Liver Disease (MELD) scores, albumin levels, platelet counts, and liver function biochemical tests [11,12,13]. Routine laboratory tests are widely available, and clinical scores are easy to calculate, aiding clinical management.

However, clinical scores based on laboratory data have low specificity for esophageal varices, necessitating subsequent endoscopic or imaging confirmation. Hemorrhage risk quantification based on these scores may underestimate the risk and complexity of varices and potential bleeding [14].

The integration of AI-based systems into the management of esophageal varices holds significant potential for improving diagnosis, individualized risk stratification, and optimizing therapeutic strategies for each patient. The main advantages of AI systems lie in their ability to process large volumes of data rapidly and to provide recommendations by integrating various patient data and information. Advances in AI algorithms have led to the development of machine learning (ML) systems, which recognize patterns in datasets, and deep learning (DL), a subset of ML that performs automatic classifications based on computed data [15].

The aim of this systematic review is to present, in a structured and rigorous manner, the various applications of AI-based systems in the management of esophageal varices. This includes algorithms that analyze clinical scores and laboratory parameters, endoscopic images, and imaging studies such as CT scans.

## 2. Materials and Methods

### 2.1. Search Strategy

This systematic review was developed and written following the latest Preferred Reporting Items for Systematic Reviews and Meta-Analyses (PRISMA) protocol from 2020, adhering to criteria for identifying, selecting, analyzing the quality of studies, assessing risk of bias, and synthesizing the data coherently [16].

To conduct this study, articles to be included were searched using the PubMed, EMBASE, Wiley, and Cochrane Library databases from their inception up to June 2024.

The search was performed using the following combinations of terms: (“artificial intelligence” OR “AI” OR “machine learning” OR “deep learning” OR “computer-aided diagnosis” OR “neural networks” OR “artificial neural networks” OR “computer-aided management”) AND (“management” OR “diagnosis” OR “analysis” OR “evaluation” OR “identification” OR “risk assessment”) AND (“esophageal varices” OR “esophageal bleeding” OR “esophageal veins” OR “esophageal hemorrhage” OR “variceal bleeding” OR “variceal hemorrhage”).

### 2.2. Inclusion and Exclusion Criteria

This systematic review included clinical studies published in English up to June 2024 that analyzed adult cirrhotic patients with esophageal varices and assessed the effectiveness of AI systems in managing these varices.

Systematic reviews, meta-analyses, studies involving pediatric populations, preclinical studies, articles published in languages other than English, abstracts, conference reports or proceedings, case reports, editorials, and letters to the editor were subsequently excluded from the study. Additionally, studies that evaluated the effectiveness of AI systems in managing both esophageal and gastric varices and reported results collectively, rather than by subgroup, were excluded. This systematic review focused specifically on esophageal varices.

### 2.3. Study Selection and Data Extraction

Selected studies that met the inclusion and exclusion criteria were analyzed, and the following data were collected: authors’ names, publication year, number of patients or images and their relevant characteristics, the AI algorithm used, and the main outcomes reported.

A total of 37 studies were identified in the PubMed and EMBASE databases. Of these, 26 articles underwent the verification and selection process, and ultimately, 14 studies that met the specified criteria were included.

**Figure 1:**
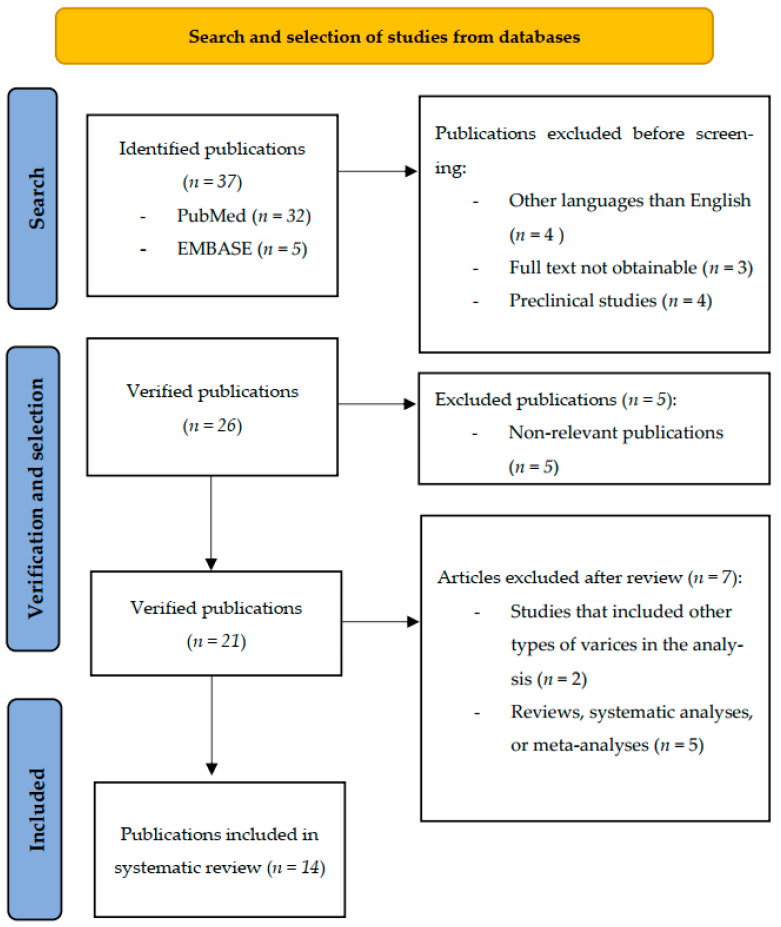
PRISMA flowchart illustrating the process of identification, screening, selection, and inclusion of studies in the systematic review.

The flow diagram of the identification, verification, selection, and inclusion process is detailed in Figure 1.

### 2.4. Quality Assessment

Two investigators (V.I. and A.I.) conducted the quality assessment of the included studies using the Quality Assessment of Diagnostic Accuracy Studies (QUADAS-2) tool. This tool evaluates the risk of bias and concerns regarding the applicability of diagnostic tests across four domains: patient selection, index test, reference standard, and flow and timing. Each domain was analyzed through a set of eleven questions, with responses categorized as “Yes”, “No”, “Not applicable”, or “Unclear”. Both risk of bias and concerns about applicability were systematically assessed. Any disagreements between the two evaluators were resolved through discussion, and the quality ratings did not affect the inclusion of studies in the systematic review.

## 3. Results

Of the 14 selected studies, 7 analyzed the performance of AI algorithms in the management of esophageal varices based on laboratory parameters and clinical scores, 4 studies analyzed AI systems and EGD images, and 3 articles used AI systems for the analysis of CT images.

### 3.1. AI, Laboratory Parameters, and Clinical Scores

The publications that used AI algorithms and clinical data for better stratification of variceal hemorrhage risk, as well as for more accurate calculation of survival after a hemorrhagic episode, are summarized in Table 1.

Bayani et al. conducted a study to enhance predictive methods by identifying the most influential factors for predicting esophageal varix (EV) grades in patients with cirrhosis. Ensemble learning methods (CatBoost and XGB classifier) were used to determine the most important predictors of EV grades based on 26 laboratory and clinical features from 490 patients with cirrhosis. The CatBoost model had a precision of 1 in predicting all targets, while the XGB classifier had an average accuracy of 0.92 and average precision of 0.84, with a better performance for predicting grades 0 and 1. The most significant variables used for the best-performing model (Catboost) were the Child–Turcotte–Pugh (CTP) score, WBC, and vitamin K levels, alongside the INR, MCV, PT, and age [17].

A subsequent study from the same team analyzed another four algorithms: random forest (RF), artificial neural network (ANN), support vector machine (SVM), and logistic regression (LR). For grade 0, ANN and SVM had a precision of 1 and RF had the highest F1-score (0.92). For grade 1, RF had a precision of 1, ANN and SVM had a precision of 0.96 and 0.95, and the highest F1-score of 0.97 was achieved by RF, ANN, and SVM. For grade 2, RF was the best prediction model, with 1 for all measures, and SVM was the second best, with a precision of 0.97 and F1-score of 0.99. RF also performed best for grade 3, also with 1 for all measures, with ANN being the second best, with a precision of 1 and an F1-score of 0.97. Regarding recall, RF was the best performing for grades 0, 2, and 3 with a recall of 1, while SVM performed best for grade 1 with a recall of 1. RF had the best results and highest area under the curve (AUC) [18].

A prospective study of 347 patients (238 in the training cohort and 109 in the validation cohort) with screening esophagogastroduodenoscopies (EGDs) tested the accuracy of the random forest algorithm EVendo. The most important variables used for the score development were INR, AST, total bilirubin, presence of clinical ascites, BUN, and hemoglobin for the presence of EVs and presence of clinical ascites, hemoglobin, platelet count, AST, BUN, and INR for the presence of varices that needed treatment (VNT). On the training cohort, EVendo had an AUC of 0.84 (0.79–0.89) for EVs and 0.74 (0.67–0.82) for VNT. A score of ≤3.90 had a sensitivity of 0.951 and 0.946 and specificity of 0.519 and 0.377 for identifying no EVs or varices not needing treatment (VNNT), potentially sparing 30.3% of EGDs with a 4.2% (1.3–12.4%) risk of missing VNT. On the validation cohort, EVendo had a comparable AUC of 0.82 (0.74–0.91) for EV and 0.75 (0.66–0.84) for VNT. For the same score, the sensitivity was 0.923 and 1 and the specificity was 0.659 and 0.493 for no EVs/VNNT, potentially sparing 31.2% of EGDs with a 1.1% (0.1–7.4%) risk of missing VNT. The negative predictive value was 1 for ruling out VNT compared to 0.958 in the training cohort. Internal validation was used for further validation of the score, obtaining similar results [19].

Hou et al. tried predicting the esophagogastric variceal bleeding (EGVB) risk in 1100 patients (999 patients in the training cohort and 101 patients in the validation cohort) with liver cirrhosis using an ANN and 12 independent clinical and laboratory factors. The AUC of the ANN model was 0.959, significantly higher than the 0.669 of the North Italian Endoscopic Club (NIEC) and 0.725 of the revised North Italian Endoscopic Club (Rev-NIEC) indices [20].

A study conducted on 2794 patients (1283 patients in the real-world cohort and 1511 patients in the external validation cohort) developed an ML-based model called ML EGD for excluding high-risk varices (HRVs) and reducing the number of EGDs performed. The most important variables used were liver stiffness, platelet count, and total bilirubin. ML EGD could potentially reduce 607 (52.6%) EGDs, with the risk of missing 3.6% of HRVs in the training cohort and 75 (58.1%) EGDs and 1.4% HRVs in the validation cohort. The algorithm was externally validated with two test cohorts consisting of 1511 patients, obtaining 506 spared EGDs (52.4%) out of 966 total EGDs with 2.8% missed HRVs, and 224 spared EGDs (41.1%) out of 545 total EGDs with 3.1% missed HRVs. ML EGD reduced more EGDs in all cohorts compared to the Baveno IV criteria (52.6% vs. 29.4% in the training cohort, 58.1% vs. 44.2% in the validation cohort, 52.4% vs. 26.5% in test cohort 1 and 41.1% vs. 21.1% in test cohort 2) [21].

Procopet et al. evaluated the accuracy of ANNs, six serum scores, and liver stiffness in the diagnosis of cirrhosis, clinically significant portal hypertension, and esophageal varices using data from 202 patients (158 patients in the training cohort and 44 patients in the validation cohort). The ANNs used on the validation cohort, including liver stiffness, had a high diagnostic accuracy (>0.8) for all three objectives but were not statistically significant compared to only liver stiffness. The best non-invasive test was liver stiffness. The Fibrosis-4 and Lok scores were the most accurate out of the serum tests/scores [13].

A study performed on 124 cirrhotic patients tested the viability of an ML model in predicting short- and long-term survival and the outcomes of acute variceal bleeding. The s of the machine learning model were 0.87, 0.85 and 0.76 for 1, 3, and 12 months for the entire study population and 0.91, 0.88 and 0.91 for bleeding patients, outperforming the standard CTP score (0.75, 0.77, 0.69) and Model for End-Stage Liver Disease with added sodium (MELD-Na score) (0.74, 0.73, 0.68) [22].

### 3.2. AI and Endoscopy Images

The main characteristics of the studies that analyzed the performance of AI-based systems in the management of esophageal varices using images obtained from EGD are illustrated in Table 2.

A retrospective analysis analyzed whether XGBoost (Extreme Gradient Boosting Algorithm) could predict future variceal bleeding (VB) better than endoscopic classification alone on 828 patients with compensated advanced chronic liver disease (cACLD) with EVs. The most important variables used for the ML model were endoscopic classification and liver stiffness measurement. XGBoost’s accuracy in predicting future VB was 0.987 (0.974–0.995) on the training cohort (497 patients), 0.937 (0.888–0.972) on the internal validation cohort (149 patients), and 0.857 (0.821–0.905) on the external validation cohort (182 patients), surpassing the accuracy of endoscopic classification alone [0.589 (0.555–0.623)]. Patients classified as high-risk by both the endoscopy and ML model had 1-year and 3-year bleeding rates of 31–43% and 64–85%, while those classified as low-risk by both methods had 1-year and 3-year bleeding rates of 0–1.6% and 0–3.4% [23].

Chen et al. trained and tested ENDOANGEL, a real-time deep convolutional neural network system (DCNN), to diagnose gastroesophageal varices and predict their rupture risk using 8566 images of endoscopic gastroesophageal varices from 3021 patients and 6152 images of normal esophagus/stomach from 3168 patients. On the test dataset, ENDOANGEL had a sensitivity of 0.995 and specificity of 0.994 on the EV test, with an accuracy of 0.995 for detecting EVs, 0.925 for size measurement, 0.860 for red color signs (RC), 0.634 for categorizing EV form, 0.8 for identifying the color of EVs, 0.922 for EV bleeding signs, and 0.709 for mucosal findings. When compared to endoscopists in the man–machine image contest, there was a statistically significant difference regarding EV detection (0.97 vs. 0.939), RC (0.842 vs. 0.735), mucosal findings (0.615 vs. 0.461), and red spots (0.853 vs. 0.775). Furthermore, the average time for analyzing one image was 0.13 s for ENDOANGEL and 18.75 s for endoscopists. On the other hand, there was no statistically significant difference in identifying EVs’ size, form, and bleeding signs. ENDOANGEL also performed better in treatment follow-up and total treatment suggestions for EVs, while there was no difference in prophylactic therapy of EVs and EVs that do not require treatment. On the video dataset, per image, ENDOANGEL had an accuracy of 0.969 for detecting EVs, 0.905 for EV size, and 0.836 for RC. On the video dataset, per patient, ENDOANGEL had an accuracy of 0.973 for detecting EVs, 0.956 for EV size, and 0.913 for RC; and 0.966 for detecting GVs (gastric varices), 0.958 for GV size, and 0.931 for red spots [24].

A retrospective cohort study investigated the use of automated multimodal machine learning (MMML) for predicting EV bleeding. The pretraining was performed using 4000 cardia endoscopic images of both esophagitis and normal cardia. Initially, there were 810 images of EVs; after image augmentation, 2000 images (1000 control and 1000 bleeding) and 400 images (200 control vs. 200 bleeding) were obtained for the training and validation datasets, from 341 patients. The accuracies of the five deep learning models trained were 0.868, 0.848, 0.808, 0.763, and 0.758 for EfficientNet, Xception, ConvMixer, ResNet, and ViT. The highest-performing model, EfficientNet, had a recall, specificity, and F1-score of 0.845, 0.885, and 0.864, significantly higher than the others. The performance of six MMML models compared to four clinical indexes was evaluated on the training, validation, and test datasets. The stacking model was the highest-performing one, having an AUC of 0.975, accuracy of 0.932, sensitivity of 0.952, specificity of 0.924, recall of 0.952, precision of 0.816, and F1-score of 0.879 on the test dataset, outperforming the clinical indexes with AUCs of 0.686 (CTP), 0.680 (MELD), 0.739 [aspartate aminotransferase-to-platelet ratio index (APRI)], and 0.703 [Fibrosis-4 (FIB-4)] [25].

Another retrospective cohort study also analyzed the use of deep learning (DL) for predicting EV bleeding at 12 months. Totals of 2000 (1000 control and 1000 bleeding) and 400 (200 control and 200 bleeding) images were obtained for the training and validation datasets from 675 images in the beginning. Out of the six models, EfficientNet was the highest-performing one, achieving an accuracy of 0.992, recall of 0.99, precision of 0.993, and F1-score of 0.991 on the training dataset; accuracy of 0.91, recall of 0.9, precision of 0.918, and F1-score of 0.909 on the validation dataset; and accuracy of 0.893, recall of 0.87, precision of 0.911, and F1-score of 0.89 on the test dataset, outperforming the two endoscopists. When EfficientNet was combined with the two endoscopists, they achieved the highest performance, with an accuracy of 0.938 and 0.908, recall of 0.92 and 0.875, precision of 0.953 and 0.936, and F1-score of 0.936 and 0.904. One of the two endoscopists alone had a higher recall rate than EfficientNet alone [26].

### 3.3. AI and CT Scans

In total, three studies were identified that analyzed the performance of AI-based systems in the management of esophageal varices using CT imaging. These are summarized in Table 3.

Lee et al. worked to develop an index that combines liver and spleen volumes and clinical factors that identifies varices with high risk in 419 patients with compensated cirrhosis. The cutoff value for spleen volume/platelet ratio was determined as being >3.78, resulting in a sensitivity of 0.8 and specificity of 0.744 in the derivation cohort and a sensitivity of 0.694 and specificity of 0.785 in the validation cohort. The cutoff value of >1.63 detected all high-risk varices. Using the two cutoff values, the patients were categorized as low-risk (118 patients, 28.2%), intermediate-risk (162 patients, 38.7%), or high-risk (139 patients, 33.2%), with a cumulative 5-year incidence of VB of 0%, 1%, and 12%. The two readers categorized the patients as low-risk (314 patients, 74.9% and 297 patients, 70.9%), intermediate-risk (63 patients, 15% and 64 patients, 15.3%), or high-risk (42 patients, 10% and 58 patients, 13.8%), with VB risks of 1% and 0.7% for low-risk patients, 8.5% and 8.3% for intermediate-risk patients, and 23.9% and 19.4% for high-risk patients [27].

A retrospective–prospective single-center multi-cohort study trained and tested an ML-based radiomic model to detect high-bleeding-risk esophageal varices (HREVs) on 796 patients (391 patients in the training and internal validation cohort and 405 patients in the external validation cohort) and 2358 images. For mild EVs, the model had an AUC of 0.943, sensitivity of 0.863, specificity of 0.763, and accuracy of 0.841 on the training dataset; AUC of 0.732, sensitivity of 0.773, specificity of 0.763, and accuracy of 0.705 on the internal validation dataset; and AUC of 0.654, sensitivity of 0.773, specificity of 0.632, and accuracy of 0.641 on the external validation dataset. When applied for HREVs, the model had an AUC of 0.983, sensitivity of 0.948, specificity of 0.977, and accuracy of 0.965 on the training dataset; AUC of 0.834, sensitivity of 0.916, specificity of 0.969, and accuracy of 0.947 on the internal validation dataset; and AUC of 0.736, sensitivity of 0.69, specificity of 0.762, and accuracy of 0.743 on the external validation dataset. The model had a better performance than the Baveno VI criteria, which only had an accuracy of 0.629, sensitivity of 0.813, and specificity of 0.149, and than the expanded Baveno VI criteria, with an accuracy of 0.647, sensitivity of 0.752, and specificity of 0.372 [28].

A retrospective study performed on 330 cirrhotic patients with acute variceal bleeding (AVB) trained and tested an ML-based model for predicting the risk of hospital-based intervention or death. The model had an AUC of 0.9, sensitivity of 0.829, and specificity of 0.8 on the training dataset; AUC of 0.782, sensitivity of 0.809, and specificity of 0.625 on the internal test dataset; and AUC of 0.789, sensitivity of 0.803, and specificity of 0.5 on the external test dataset. The results were better compared to six clinical scores, which only had AUCs of 0.517, 0.532, 0.534 (admission Rockall score); 0.642, 0.730, 0.631 (Glasgow–Blatchford score); 0.687, 0.585, 0.582 (AIMS65); 0.751, 0.507, 0.589 (CTP score); 0.65, 0.554, 0.515 (MELD score); and 0.753, 0.62, 0.52 (albumin–bilirubin score) [29].

### 3.4. Quality Assessment of the Included Studies

The detailed analysis of all included studies can be found in Supplementary Table S1.

All studies appropriately enrolled patients either consecutively or randomly, avoiding case-control designs, with one study being marked as potentially with a high risk of bias in the patient selection aspect, due to unclear or not specified patient exclusion criteria [18]. Concerns regarding the applicability were low across all studies in this aspect.

The blinding of index test interpretation was unclear in several studies, specifically, these studies did not clarify whether the index test results were interpreted independently of the reference standard, thus, the risk of bias exists [17,18,20,23,29]. Across the reference standard, all studies likely classified the target condition correctly and ensured that the reference standard results were interpreted without knowledge of the index test results, maintaining a low risk of bias, with no concerns regarding the applicability being noted.

In the flow and timing domain, most studies observed an appropriate interval between the index test and the reference standard, ensuring that all patients received and were included in the analysis, with the exception of one study, in which a noticeable number of patients were not included in the final analysis [23]. Moreover, the appropriateness of the interval was unclear for several studies, marking them with a risk of bias [17,18,20].

## 4. Discussion

AI systems are increasingly utilized in many aspects of modern medicine. ML algorithms enable the rapid evaluation of substantial volumes of information with exhaustive analysis. Consequently, these systems can significantly support clinicians in managing esophageal varices by analyzing and quantifying the risk of developing esophageal varices and subsequent variceal hemorrhage, based on endoscopic images, clinical parameters, and CT images.

The Baveno VII consensus emphasizes the importance of combining individual prognostic factors according to their significance for better risk stratification of each patient, using predictive methods based on CTP and MELD scores [30]. Additionally, the Baveno VI consensus recommends assessing liver stiffness through elastography and platelet count to determine the need for EGD screening for esophageal varices. Patients with liver stiffness <20 kPa and platelet count >150 × 10^9^/L have a low risk of developing esophageal varices [12]. The study by Procopet et al., which analyzed the efficacy of ANN in managing liver cirrhosis and associated complications, confirmed that liver stiffness assessment is the most important non-invasive parameter for evaluating the risk of developing esophageal varices. FIB-4 and Lok scores also demonstrated good accuracy [13].

The main disadvantages of EGD examination include the invasiveness of the method and significant interobserver variability in the endoscopic technique, image analysis, diagnosis, and classification of esophageal varices. Studies using AI algorithms to analyze endoscopic images which directly compared these results with human experts found the AI models to have better diagnostic accuracy compared to endoscopists [24,26]. Another advantage of AI algorithms is the significant reduction in examination time (with the mean examination time per image in the study by Chen et al. being only 0.13 s compared to 18.75 s for human experts) [24].

Integrating AI into endoscopy suites facilitates continuous and real-time monitoring of procedures, flagging potential issues or lesions that might escape the human eye. This not only improves patient safety but also enhances the efficient use of medical resources. Moreover, AI can learn and adapt after each procedure, becoming increasingly precise and contributing to the continuous development of medical practices. Thus, combining human expertise with advanced AI capabilities leads to more accurate and faster endoscopic diagnosis, significantly improving patient outcomes.

An illustrative example is the study by Hong et al., where the DL algorithm achieved better accuracy compared to two human experts evaluating endoscopic images. However, when assisted by the AI program, the diagnostic accuracy of the experts in classifying esophageal varices increased by 17.3% and 19% [26].

This trend has also been confirmed by other studies in the field of gastrointestinal diseases, such as colonic adenomas and Barrett’s esophagus [31,32,33,34]. In the study conducted by Ainechi et al., less experienced endoscopists benefitted from real-time AI assistance, increasing the sensitivity of colonic polyp detection from 86.3% to 91.7% [34]. Similarly, the polyp detection rate increased from 34% to 38.7% in Lou et al.‘s study when colonoscopy was performed with AI assistance [32]. The detection rate of colonic polyps also improved for experienced endoscopists [31].

Moreover, endoscopic ultrasound (EUS) exploration of the digestive system has been steadily gaining popularity, mainly in the diagnosis and staging of esophageal, gastric, pancreatic, and colorectal cancers [35,36,37,38]. AI algorithms, particularly ML and DL systems, have demonstrated promising results in diagnosing subepithelial lesions and early gastric and esophageal cancers, as well as pancreatic diseases [37]. Thus, AI-aided EUS has the potential to reduce interobserver variability, enhance lesion detection and characterization, as well as improve patients’ outcomes and reduce the need of reintervention and other procedures due to inconclusive results [35,36,37]. A meta-analysis of AI-aided EUS for subepithelial lesions revealed high diagnostic performance, with a combined sensitivity of 0.92, specificity of 0.80, and area under the curve of 0.92, as well as superior sensitivity (0.93 vs. 0.71), specificity (0.81 vs. 0.79), and AUC (0.94 vs. 0.75) compared to the human experts [38]. Another meta-analysis showed that AI-assisted EUS significantly enhanced the detection rate and helped distinguish between pancreatic ductal adenocarcinomas and other solid tumors, with the highest accuracy being recorded for the contrast-enhanced EUS and EUS-guided elastography [39].

The integration of EGD, CT imaging, laboratory parameters, and clinical scores is crucial for the proper management of patients with esophageal varices. CT imaging provides essential details about liver anatomy, the portal venous system, and helps identify signs of portal hypertension. Clinical parameters, such as platelet count, albumin and bilirubin levels, and subsequent classification of the patient into a CTP or MELD category, are vital for guiding therapeutic decisions. AI can assist in integrating and analyzing all these data to provide precise evaluations and better patient management.

However, most studies conducted so far on the applications of AI-based systems in the management of esophageal varices have been retrospective. Multicentric clinical trials with a significant number of patients, conducted over longer periods, and evaluating the potential of these technologies in real-time endoscopies are necessary to formulate recommendations for the use of AI algorithms in daily medical practice.

Integrating these technologies is not without challenges, ranging from possible reluctance of medical staff to adopt such technologies to the need for close collaboration between healthcare and technology professionals to ensure the proper functioning of these algorithms and devices. Additionally, it is necessary to develop appropriate training programs for medical staff and continuously update medical techniques as algorithms and technologies improve.

When applying AI systems in medicine, it is essential to acknowledge several limitations that could impact their effectiveness and reliability. One major concern is the issue of model interpretability. The lack of transparency can be problematic in medical contexts, where understanding the reasoning behind a diagnosis or treatment recommendation is crucial for gaining trust, ensuring patient safety, and adhering to ethical standards. Without clear interpretability, there is a risk that AI could make decisions based on spurious correlations or biases present in the training data, leading to potentially harmful outcomes. Explainable AI techniques are being developed to address this, including model-based and post hoc explanation methods [40].

Another critical limitation of AI in medicine is the tendency for models to overfit and the challenges related to generalizability. Overfitting occurs when a model performs exceptionally well on the training data but fails to generalize to new, unseen data. This can happen due to the model learning noise or irrelevant patterns specific to the training dataset rather than the underlying medical principles [41]. Consequently, AI systems may produce inaccurate predictions or recommendations when applied to different patient populations or clinical settings not represented in the training data. Ensuring that AI models are robust and generalizable across diverse medical contexts requires careful validation, the use of large and diverse datasets, and ongoing monitoring to prevent these issues from compromising the quality of care.

The financial aspect is also critical. Since new technologies are still in their early stages, the general perception among both the public and many professionals is that they are currently very expensive and not easily accessible to hospitals, particularly smaller and medium-sized centers. Implementing these technologies indeed involves initial costs related to acquiring and integrating the necessary equipment and technologies into the existing infrastructure, as well as additional training for medical staff. However, several studies analyzing the financial impact of implementing AI-based systems in gastroenterology have shown increased hospital revenues due to a higher number of patients and interventions performed [42,43,44].

Furthermore, AI algorithms facilitate earlier diagnosis of many gastrointestinal diseases associated with significant morbidity and mortality, such as colorectal and gastric cancer [42,43,44,45]. Earlier diagnosis and personalized management, both facilitated by AI algorithms, lead to significant improvements in life expectancy, translating into additional revenue and significant social benefits. These include preventing the progression of disabling diseases, reducing life-threatening complications, and decreasing the number of hospitalization days and long-term treatments [42].

Thus, the assistance these programs can provide to clinicians in daily practice can translate into more efficient use of medical resources, optimizing the economic efficiency of medical institutions.

## 5. Conclusions

The aim of this study was to systematically analyze AI-based applications in the management of esophageal varices, bringing together 14 studies that evaluated AI algorithms in endoscopy, CT imaging, laboratory analyses, and clinical scores. In most studies, AI applications were trained and subsequently tested on both internal and external databases, and their accuracy was comparable to, or in some cases even superior to, that of human experts.

Implementing AI systems in medicine has the potential to provide personalized and high-quality patient care, while also assisting clinicians by analyzing large amounts of data in a short time and integrating multiple pieces of information for more efficient, safe, and personalized diagnosis and treatment plans. This approach also reduces the risk of medical errors and the costs associated with patient care.

The evaluation of such technologies and the decision to implement them in daily medical practice require prospective, multicentric studies and a comprehensive analysis by international experts to quantify the large-scale applicability of AI-based systems.

## Figures and Tables

**Table 1:** Studies analyzing AI and laboratory parameters.

Author (Year)	Type of Algorithm	Number of Patients	Main Findings
Bayani et al. (2022) [17]	EL	490 patients 236 with EVs 5-fold cross-validation	CatBoost: Precision: 1 XGB classifier: Precision: 0.84 Accuracy: 0.92
Bayani et al. (2022) [18]	ML: RF ANN SVM LR	490 patients 236 with EVs 5-fold cross-validation	RF: Best results and best AUC Recall: 1 for grade 0, 2, and 3 EVs Precision: 1 for grade 1, 2, and 3 EVs F1-score: 1 for grade 2 and 3 EVs ANN: Precision: 1 for grade 0 and 3 EVs SVM: Recall: 1 for grade 1 and 3 EVs Precision: 1 for grade 0 and 2 EVs
Dong et al. (2019) [19]	RF	347 patients 238 in the training cohort 109 in the validation cohort	For the score determined (≤3.90): In the training cohort: AUC: 0.84 (0.79–0.89) for EVs AUC: 0.74 (0.67–0.82) for VNT In the validation cohort: AUC: 0.82 (0.74–0.91) for EVs AUC: 0.75 (0.66–0.84) for VNT
Hou et al. (2023) [20]	ANN	1100 patients 999 in the training cohort 101 in the validation cohort	AUC: 0.959 Outperformed: NIEC index (AUC: 0.669) Rev-NIEC index (AUC: 0.725)
Huang et al. (2023) [21]	ML	2794 patients 1283 patients in the real-world cohort 1154 in the training cohort 129 in the internal validation cohort 1511 patients in the external validation cohort 966 test cohort 1 545 test cohort 2	In the training cohort: 52.6% spared EGDs 3.6% missed HRVs In the validation cohort: 58.1% spared EGDs 1.4% missed HRVs In the test cohorts: 52.4% spared EGDs 2.8% missed HRVs 41.1% spared EGDs 3.1% missed HRVs Better performance than Baveno IV criteria in all cohorts.
Procopet et al. (2015) [13]	ANN	202 patients 69 with EVs 158 in the training cohort 44 in the validation cohort	High diagnostic accuracy (>0.8). Not statistically significant compared to only liver stiffness. Liver stiffness was the best non-invasive test. Fibrosis-4 and Lok scores were the most accurate out of the serum tests/scores.
Simsek et al. (2021) [22]	ML	124 patients 80 with EVs	For the entire population: AUC: 0.87 at 1 month AUC: 0.85 at 3 months AUC: 0.76 at 12 months For bleeding patients: AUC: 0.91 at 1 month AUC: 0.88 at 3 months AUC: 0.91 at 12 months Better performance than: CTP score (AUCs: 0.75, 0.77, 0.69) MELD-Na score (AUCs: 0.74, 0.73, 0.68)

EL: ensemble learning; ML: machine learning; RF: random forest; ANN: artificial neural network; SVM: support vector machine; LR: logistic regression; EVs: esophageal varices; VNT: varices that needed treatment; NIEC: North Italian Endoscopic Club; Rev-NIEC: revised North Italian Endoscopic Club; EGD: esophagogastroduodenoscopy; HRVs: high-risk varices; CTP: Child–Turcotte–Pugh; MELD-Na: Model for End-Stage Liver Disease with added sodium.

**Table 2:** AI systems and endoscopy images.

Author (Year)	Type of Algorithm	Number of Patients	Main Findings
Agarwal et al. (2021) [23]	ML	828 patients 497 in the training cohort 149 patients in the internal validation cohort 182 patients in the external validation cohort	On the training cohort: Accuracy: 0.987 On the internal validation cohort: Accuracy: 0.937 On the external validation cohort: Accuracy: 0.857 Better performance than endoscopic classification alone (Accuracy: 0.589)
Chen et al. (2020) [24]	DCNNs	3021 patients 8566 endoscopic gastroesophageal varices images 3168 patients 6152 normal esophagus/stomach images	On the test dataset for EVs: Accuracy: 0.995 Sensitivity: 0.995 Specificity: 0.994 Compared to endoscopists: Statistically significant difference in: EV detection (0.97 vs. 0.939) RC (0.842 vs. 0.735) Mucosal findings (0.615 vs. 0.461) Red spots (0.853 vs. 0.775) Average time/image (0.13 s vs. 18.75 s) Treatment follow-up (EVs) Total treatment suggestion (EVs) No statistically significant difference in: Size (EVs) Form (EVs) Bleeding signs (EVs) Prophylactic therapy (EVs) EVs that do not require treatment
Wang et al. (2022) [25]	MMML	Pretraining dataset: 4000 normal/esophagitis cardia endoscopic images Initially: 810 images of EVs from 341 patients After image augmentation: Training dataset: 2000 images 1000 control 1000 bleeding Validation dataset: 400 images 200 control 200 bleeding	EfficientNet was the highest-performing DL model with: Accuracy: 0.868 Recall: 0.845 Specificity: 0.885 F1-score: 0.864 Stacking model was the highest-performing MMML model in the test dataset with: AUC: 0.975 Accuracy: 0.932 Sensitivity: 0.952 Specificity: 0.924 Recall: 0.952 Precision: 0.816 F1-score: 0.879 It performed better than all clinical indexes: AUC: 0.686 (CTP) AUC: 0.680 (MELD) AUC: 0.739 (APRI) AUC: 0.703 (FIB-4)
Hong et al. (2023) [26]	DL	Initially: 675 images of EVs After image augmentation: Training dataset: 2000 images 1000 control 1000 bleeding Validation dataset: 400 images 200 control 200 bleeding	EfficientNet was the highest-performing DL model and performed better than the 2 endoscopists with: On the training dataset: Accuracy: 0.992 Recall: 0.99 Precision: 0.993 F1-score: 0.991 On the validation dataset: Accuracy: 0.91 Recall: 0.9 Precision: 0.918 F1-score: 0.909 On the test dataset: Accuracy: 0.893 Recall: 0.87 Precision: 0.911 F1-score: 0.89 Highest performance achieved by combining the 2 endoscopists and EfficientNet with: Accuracy: 0.938 and 0.908 Recall: 0.92 and 0.875 Precision: 0.953 and 0.936 F1-score: 0.936 and 0.904

ML: machine learning; DCNNs: deep convolutional neural networks; EVs: esophageal varices; RC: red color signs; MMML: multimodal machine learning; DL: deep learning; CTP: Child–Turcotte–Pugh; MELD: Model for End-Stage Liver Disease; APRI: aspartate aminotransferase-to-platelet ratio index; FIB-4: Fibrosis-4.

**Table 3:** AI systems and CT scan images.

Author (Year)	Type of Algorithm	Number of Patients	Main Findings
Lee et al. (2020) [27]	DL	419 patients	Determined cutoff value for spleen volume/platelet ratio: >3.78 On the derivation cohort: Sensitivity: 0.8 Specificity: 0.744 On the validation cohort: Sensitivity: 0.694 Specificity: 0.785 Cutoff value that detected all high-risk varices: >1.63 Using the two cutoff values, patients categorized as low-, intermediate-, and high-risk with a cumulative 5-year incidence of variceal bleeding of 0%, 1%, and 12%.
Yan et al. (2022) [28]	ML	796 patients 391 in the training and internal validation cohort 405 in the external validation cohort 2358 images	For mild EVs: On the training dataset: AUC: 0.943 Sensitivity: 0.863 Specificity: 0.763 Accuracy: 0.841 On the internal validation dataset: AUC: 0.732 Sensitivity: 0.773 Specificity: 0.763 Accuracy: 0.705 On the external validation dataset: AUC: 0.654 Sensitivity: 0.773 Specificity: 0.632 Accuracy: 0.641 For HREVs: In the training dataset: AUC: 0.983 Sensitivity: 0.948 Specificity: 0.977 Accuracy: 0.965 On the internal validation dataset: AUC: 0.834 Sensitivity: 0.916 Specificity: 0.969 Accuracy: 0.947 On the external validation dataset: AUC: 0.736 Sensitivity: 0.69 Specificity: 0.762 Accuracy: 0.743 Model performed better than Baveno VI and expanded Baveno VI criteria.
Gao et al. (2023) [29]	ML	330 patients 5-fold cross-validation	On the training dataset: AUC: 0.9 Sensitivity: 0.829 Specificity: 0.8 On the internal test dataset: AUC: 0.782 Sensitivity: 0.809 Specificity: 0.625 On the external test dataset: AUC: 0.789 Sensitivity: 0.803 Specificity: 0.5 Model performed better than clinical scores: ARS (AUCs: 0.517, 0.532, 0.534) GBS (AUCs: 0.642, 0.730, 0.631) AIMS65 (AUCs: 0.687, 0.585, 0.582) CTP (AUCs: 0.751, 0.507, 0.589) MELD (AUCs: 0.65, 0.554, 0.515) ALBI (AUCs: 0.753, 0.62, 0.52)

DL: deep learning; ML: machine learning; EVs: esophageal varices; ARS: admission Rockall score; GBS: Glasgow–Blatchford score; CTP: Child–Turcotte–Pugh score; MELD: Model for End-Stage Liver Disease score; ALBI: albumin–bilirubin score.

## Data Availability

Data contained within the article.

## Supplementary Materials

The following supporting information can be downloaded at: https://www.mdpi.com/article/10.3390/jpm14091012/s1, Table S1: QUADAS-2 tool for evaluating the methodological quality of included studies.

## References

[1] Yamada T., Alpers D.H., Kalloo A.N., Kaplowitz N., Owyang C., Powell D.W. (2009). Textbook of Gastroenterology.

[2] Lesmana C.R.A., Raharjo M., Gani R.A. (2020). Managing liver cirrhotic complications: Overview of esophageal and gastric varices. Clin. Mol. Hepatol..

[3] Garcia-Tsao G., Sanyal A.J., Grace N.D., Carey W., Shuhart M.C., Davis G.L., Practice Guidelines Committee of the American Association for the Study of Liver Diseases, The Practice Parameters Committee of the American College of Gastroenterology (2007). Prevention and management of gastroesophageal varices and variceal hemorrhage in cirrhosis. Hepatology.

[4] Gralnek I.M., Duboc M.C., Garcia-Pagan J.C., Fuccio L., Karstensen J.G., Hucl T., Jovanovic I., Awadie H., Hernandez-Gea V., Tantau M. (2022). Endoscopic diagnosis and management of esophagogastric variceal hemorrhage: European Society of Gastrointestinal Endoscopy (ESGE) Guideline. Endoscopy.

[5] Frenette C.T., Kuldau J.G., Hillebrand D.J., Lane J., Pockros P.J. (2008). Comparison of esophageal capsule endoscopy and esophagogastroduodenoscopy for diagnosis of esophageal varices. World J. Gastroenterol..

[6] Lipp M.J., Broder A., Hudesman D., Suwandhi P., Okon S.A., Horowitz M., Clain D.J., Friedmann P., Min A.D. (2011). Detection of esophageal varices using CT and MRI. Dig. Dis. Sci..

[7] Borhani A., Luu H., Mohseni A., Xu Z., Shaghaghi M., Tolosa C., Attari M.M.A., Madani S.P., Shahbazian H., Khoshpouri P. (2024). Screening for exclusion of high-risk bleeding features of esophageal varices in cirrhosis through CT and MRI. Clin. Imaging.

[8] Mifune H., Akaki S., Ida K., Sei T., Kanazawa S., Okada H. (2007). Evaluation of esophageal varices by multidetector-row CT: Correlation with endoscopic “red color sign”. Acta Med. Okayama.

[9] Meng D., Wei Y., Feng X., Kang B., Wang X., Qi J., Zhao X., Zhu Q. (2021). CT-Based Radiomics Score Can Accurately Predict Esophageal Variceal Rebleeding in Cirrhotic Patients. Front. Med..

[10] Paternostro R., Reiberger T., Bucsics T. (2019). Elastography-based screening for esophageal varices in patients with advanced chronic liver disease. World J. Gastroenterol..

[11] Pateu E., Oberti F., Calès P. (2018). The noninvasive diagnosis of esophageal varices and its application in clinical practice. Clin. Res. Hepatol. Gastroenterol..

[12] Bai W., Abraldes J.G. (2022). Noninvasive assessment oesophageal varices: Impact of the Baveno VI criteria. Curr. Opin. Gastroenterol..

[13] Procopet B., Cristea V.M., Robic M.A., Grigorescu M., Agachi P.S., Metivier S., Peron J.M., Selves J., Stefanescu H., Berzigotti A. (2015). Serum tests, liver stiffness and artificial neural networks for diagnosing cirrhosis and portal hypertension. Dig. Liver Dis..

[14] Mattos Â.Z., Schacher F.C., John Neto G., Mattos A.A. (2019). Screening for esophageal varices in cirrhotic patients—Non-invasive methods. Ann. Hepatol..

[15] Kröner P.T., Engels M.M., Glicksberg B.S., Johnson K.W., Mzaik O., van Hooft J.E., Krittanawong C. (2021). Artificial intelligence in gastroenterology: A state-of-the-art review. World J. Gastroenterol..

[16] Page M.J., McKenzie J.E., Bossuyt P.M., Boutron I., Hoffmann T.C., Mulrow C.D., Moher D. (2021). The PRISMA 2020 statement: An updated guideline for reporting systematic reviews. BMJ.

[17] Bayani A., Hosseini A., Asadi F., Hatami B., Kavousi K., Aria M., Zali M.R. (2022). Identifying predictors of varices grading in patients with cirrhosis using ensemble learning. Clin. Chem. Lab. Med..

[18] Bayani A., Asadi F., Hosseini A., Hatami B., Kavousi K., Aria M., Zali M.R. (2022). Performance of machine learning techniques on prediction of esophageal varices grades among patients with cirrhosis. Clin. Chem. Lab. Med..

[19] Dong T.S., Kalani A., Aby E.S., Le L., Luu K., Hauer M., Kamath R., Lindor K.D., Tabibian J.H. (2019). Machine Learning-based Development and Validation of a Scoring System for Screening High-Risk Esophageal Varices. Clin. Gastroenterol. Hepatol..

[20] Hou Y., Yu H., Zhang Q., Yang Y., Liu X., Wang X., Jiang Y. (2023). Machine learning-based model for predicting the esophagogastric variceal bleeding risk in liver cirrhosis patients. Diagn. Pathol..

[21] Huang Y., Li J., Zheng T., Ji D., Wong Y.J., You H., Gu Y., Li M., Zhao L., Li S. (2023). Development and validation of a machine learning-based model for varices screening in compensated cirrhosis (CHESS2001): An international multicenter study. Gastrointest. Endosc..

[22] Simsek C., Sahin H., Tekin I.E., Sahin T.K., Balaban H.Y., Sivri B. (2021). Artificial intelligence to predict overall survivals of patients with cirrhosis and outcomes of variceal bleeding. Hepatol. Forum.

[23] Agarwal S., Sharma S., Kumar M., Venishetty S., Bhardwaj A., Kaushal K., Gopi S., Mohta S., Gunjan D., Saraya A. (2021). Development of a machine learning model to predict bleed in esophageal varices in compensated advanced chronic liver disease: A proof of concept. J. Gastroenterol. Hepatol..

[24] Chen M., Wang J., Xiao Y., Wu L., Hu S., Chen S., Yi G., Hu W., Xie X., Zhu Y. (2021). Automated and real-time validation of gastroesophageal varices under esophagogastroduodenoscopy using a deep convolutional neural network: A multicenter retrospective study (with video). Gastrointest. Endosc..

[25] Wang Y., Hong Y., Zhou X., Gao X., Yu C., Lin J., Liu L., Gao J., Yin M., Xu G. (2023). Automated Multimodal Machine Learning for Esophageal Variceal Bleeding Prediction Based on Endoscopy and Structured Data. J. Digit. Imaging.

[26] Hong Y., Yu Q., Mo F., Yin M., Xu C., Zhu S., Lin J., Xu G., Gao J., Liu L. (2023). Deep learning to predict esophageal variceal bleeding based on endoscopic images. J. Int. Med. Res..

[27] Lee C.M., Lee S.S., Choi W.M., Kim K.M., Sung Y.S., Lee S., Suk H.I. (2021). An index based on deep learning-measured spleen volume on CT for the assessment of high-risk varix in B-viral compensated cirrhosis. Eur. Radiol..

[28] Yan Y., Li Y., Fan C., Zhang Y., Zhang S., Wang Z., Huang T., Ding Z., Hu K., Li L. (2022). A novel machine learning-based radiomic model for diagnosing high bleeding risk esophageal varices in cirrhotic patients. Hepatol. Int..

[29] Gao Y., Yu Q., Li X., Xia C., Zhou J., Xia T., Zhao B., Qiu Y., Zha J.-H., Wang Y. (2023). An imaging-based machine learning model outperforms clinical risk scores for prognosis of cirrhotic variceal bleeding. Eur. Radiol..

[30] De Franchis R., Bosch J., Garcia-Tsao G., Reiberger T., Ripoll C., Abraldes J.G., Albillos A., Baiges A., Bajaj J., Bañares R. (2022). Baveno VII—Renewing consensus in portal hypertension. J. Hepatol..

[31] Koh F.H., Ladlad J., Teo E.-K., Lin C.-L., Foo F.-J. (2023). Real-time artificial intelligence (AI)-aided endoscopy improves adenoma detection rates even in experienced endoscopists: A cohort study in Singapore. Surg. Endosc..

[32] Luo Y., Zhang Y., Liu M., Lai Y., Liu P., Wang Z., Xing T., Huang Y., Li Y., Li A. (2021). Artificial Intelligence-Assisted Colonoscopy for Detection of Colon Polyps: A Prospective, Randomized Cohort Study. J. Gastrointest. Surg..

[33] Meinikheim M., Mendel R., Palm C., Probst A., Muzalyova A., Scheppach M.W., Ebigbo A. (2023). Effect of AI on performance of endoscopists to detect Barrett neoplasia: A Randomized Tandem Trial. Endoscopy.

[34] Ainechi D., Misawa M., Barua I., Larsen S.L.V., Paulsen V., Garborg K.K., Aabakken L., Tønnesen C.J., Løberg M., Kalager M. (2022). Impact of artificial intelligence on colorectal polyp detection for early-career endoscopists: An international comparative study. Scand. J. Gastroenterol..

[35] Liu E., Bhutani M.S., Sun S. (2021). Artificial intelligence: The new wave of innovation in EUS. Endosc. Ultrasound.

[36] Agudo Castillo B., Mascarenhas M., Martins M., Mendes F., de la Iglesia D., Costa A.M.M.P.D., Esteban Fernández-Zarza C., González-Haba Ruiz M. (2024). Advancements in biliopancreatic endoscopy: A comprehensive review of artificial intelligence in EUS and ERCP. Rev. Esp. Enferm. Dig..

[37] Huang J., Fan X., Liu W. (2023). Applications and Prospects of Artificial Intelligence-Assisted Endoscopic Ultrasound in Digestive System Diseases. Diagnostics.

[38] Liu X.Y., Song W., Mao T., Zhang Q., Zhang C., Li X.Y. (2022). Application of artificial intelligence in the diagnosis of subepithelial lesions using endoscopic ultrasonography: A systematic review and meta-analysis. Front. Oncol..

[39] Prasoppokakorn T., Tiyarattanachai T., Chaiteerakij R., Decharatanachart P., Mekaroonkamol P., Ridtitid W., Kongkam P., Rerknimitr R. (2022). Application of artificial intelligence for diagnosis of pancreatic ductal adenocarcinoma by EUS: A systematic review and meta-analysis. Endosc. Ultrasound.

[40] Akhai S. (2023). From black boxes to transparent machines: The quest for explainable AI. SSRN.

[41] Tomsett R., Preece A., Braines D., Cerutti F., Chakraborty S., Srivastava M., Pearson G., Kaplan L. (2020). Rapid trust calibration through interpretable and uncertainty-aware AI. Patterns.

[42] Yonazu S., Ozawa T., Nakanishi T., Ochiai K., Shibata J., Osawa H., Hirasawa T., Kato Y., Tajiri H., Tada T. (2024). Cost-effectiveness analysis of the artificial intelligence diagnosis support system for early gastric cancers. DEN Open.

[43] Hassan C., Repici A., Mori Y. (2023). Cost of artificial intelligence: Elephant in the room and its cage. Dig. Endosc..

[44] Chin S.-E., Wan F.-T., Ladlad J., Chue K.-M., Centre S.E., Teo E.-K., Lin C.-L., Foo F.-J., Koh F.H. (2023). One-year review of real-time artificial intelligence (AI)-aided endoscopy performance. Surg. Endosc..

[45] Tokat M., Van Tilburg L., Koch A.D., Spaander M.C.W. (2022). Artificial Intelligence in Upper Gastrointestinal Endoscopy. Dig. Dis..

