# Supplementary material for: Applications of Artificial Intelligence-Based Systems in the Management of Esophageal Varices
Source: J Pers Med. 2024 Sep 23;14(9):1012. doi: 10.3390/jpm14091012 (PMC11433421; doi:10.3390/jpm14091012)
Supplement: Supplementary file 1 [file jpm-14-01012-s001.zip › jpm-3175179-supplementary.pdf]

**Criteria**	*Agarval et al. (2021)*	*Bayani et al. (July 2022)*	*Bayani et al. (Sept 2022)*	*Chen et al. (2020)*	*Dong et al. (2019)*	*Gao et al. (2023)*	*Hong et al. (2023)*	*Hou et al. (2023)*	*Huang et al. (2023)*	*Lee et al. (2020)*	*Procopet et al. (2015)*	*Simsek et al. (2021)*	*Wang et al. (2022)*	*Yan et al. (2022)*
**Domain 1 – Patient selection**														
**A. Risk of bias**														
Was a consecutive or random sample of patients enrolled?	No	Yes	Yes	Yes	Yes	Yes	Yes	Yes	Yes	Yes	Yes	Yes	Yes	Yes
Was a case-control design avoided?	Yes	Yes	Yes	Yes	Yes	Yes	Yes	Yes	Yes	Yes	Yes	Yes	Yes	Yes
Did the study avoid inappropriate exclusions?	Yes	Unclear	Yes	Yes	Yes	Yes	Yes	Yes	Yes	Yes	Yes	Yes	Yes	Yes
**Risk of bias assessment**	Low	High	Low	Low	Low	Low	Low	Low	Low	Low	Low	Low	Low	Low
**B. Concerns regarding applicability**	Low	Low	Low	Low	Low	Low	Low	Low	Low	Low	Low	Low	Low	Low
**Domain 2 – Index test**														
**A. Risk of bias**														
Were the index test results interpreted without knowledge of the results of the reference standard?	Unclear	Unclear	Unclear	Yes	Yes	Unclear	Yes	Unclear	Yes	Yes	Yes	Yes	Yes	Yes
If a threshold was used, was it pre-specified?	NA	Unclear	NA	No	Yes	NA	No	NA	Yes	Yes	Yes	Yes	Yes	No
**Risk of bias assessment**	High	High	High	Low	Low	High	Low	High	Low	Low	Low	Low	Low	Low
**B. Concerns regarding applicability**	Low	Low	Low	Low	Low	Low	Low	Low	Low	Low	Low	Low	Low	Low
**Domain 3 – Reference standard**														
**A. Risk of bias**														
Is the reference standard likely to correctly classify the target condition?	Yes	Yes	Yes	Yes	Yes	Yes	Yes	Yes	Yes	Yes	Yes	Yes	Yes	Yes
Were the reference standard results interpreted without knowledge of the results of the index test?	Yes	Yes	NA	Yes	Yes	Yes	NA	Yes	Yes	Yes	Yes	Yes	Yes	Yes
**Risk of bias assessment**	Low	Low	Low	Low	Low	Low	High	Low	Low	Low	Low	Low	Low	Low
**B. Concerns regarding applicability**	Low	Low	Low	Low	Low	Low	Low	Low	High	Low	Low	Low	Low	Low
**Domain 4 – Flow and timing**														
**A. Risk of bias**														
Was there an appropriate interval between index test(s) and reference standard?	Yes	Unclear	Unclear	Yes	Yes	Yes	Yes	Unclear	Yes	Yes	Yes	Yes	Yes	Yes

Did all patients receive a reference standard?	Yes	Yes	Yes	Yes	Yes	Yes	Yes	Yes	Yes	Yes	Yes	Yes	Yes	Yes
Did patients receive the same reference standard?	Yes	Yes	Yes	Yes	Yes	Yes	Yes	Yes	Yes	Yes	Yes	Yes	Yes	Yes
Were all patients included in the analysis?	No	Yes	Yes	No	Yes	Unclear	Yes	Yes	Yes	Yes	Yes	Yes	Yes	Yes
**Risk of bias assessment**	High	Low	Unclear	Low	Low	Low	Low	Low	Low	Low	Low	Low	Low	Low

Supplementary Table S1. QUADAS-2 tool for evaluating the methodological quality of included studies

NA: Not applicable
